# Organization of an Activator-Bound RNA Polymerase Holoenzyme

**DOI:** 10.1016/j.molcel.2008.09.015

**Published:** 2008-11-07

**Authors:** Daniel Bose, Tillmann Pape, Patricia C. Burrows, Mathieu Rappas, Siva R. Wigneshweraraj, Martin Buck, Xiaodong Zhang

**Affiliations:** 1 Division of Molecular Biosciences, Centre for Structural Biology, Department of Life Sciences, Faculty of Natural Sciences, Imperial College London, London SW7 2AZ, UK; 2 Division of Biology, Department of Life Sciences, Faculty of Natural Sciences, Imperial College London, London SW7 2AZ, UK; 3 Division of Investigative Science, Department of Microbiology, Faculty of Medicine, Imperial College London, London SW7 2AZ, UK

**Keywords:** DNA, PROTEINS, MICROBIO

## Abstract

Transcription initiation involves the conversion from closed promoter complexes, comprising RNA polymerase (RNAP) and double-stranded promoter DNA, to open complexes, in which the enzyme is able to access the DNA template in a single-stranded form. The complex between bacterial RNAP and its major variant sigma factor σ^54^ remains as a closed complex until ATP hydrolysis-dependent remodeling by activator proteins occurs. This remodeling facilitates DNA melting and allows the transition to the open complex. Here we present cryoelectron microscopy reconstructions of bacterial RNAP in complex with σ^54^ alone, and of RNAP-σ^54^ with an AAA+ activator. Together with photo-crosslinking data that establish the location of promoter DNA within the complexes, we explain why the RNAP-σ^54^ closed complex is unable to access the DNA template and propose how the structural changes induced by activator binding can initiate conformational changes that ultimately result in formation of the open complex.

## Introduction

Multisubunit RNA polymerases (RNAP) are the central enzymes for accessing genetic information. They are structurally and functionally conserved between Bacteria, Archaea, and Eukarya. Bacterial sigma (σ) factors, which are divided into two distinct classes, recognize promoter sequences and form holoenzymes with RNAP to ensure productive transcription. σ^70^ transcribes most housekeeping genes, whereas the major variant, σ^54^, controls transcription of genes expressed under specific environmental conditions (Buck et al., 2000; Reitzer and Schneider, 2001). The two classes of holoenzyme have contrasting properties. RNAP-σ^70^ can spontaneously isomerize to form open promoter complexes for gene transcription, whereas RNAP-σ^54^ forms a transcriptionally silent closed complex. Open complex formation by RNAP-σ^54^ requires ATP hydrolysis by activator proteins, which bind to enhancer-like sequences upstream of the promoter site (Popham et al., 1989; Sasse-Dwight and Gralla, 1988b; Wedel and Kustu, 1995; Wedel et al., 1990). σ^54^ activators are therefore also referred to as bacterial enhancer binding proteins (bEBP). This activation process resembles that of eukaryotic RNA polymerase II, in which DNA opening is accomplished by TFIIH in a reaction consuming ATP (Kim et al., 2000; Lin et al., 2005). The transition from closed to open promoter complexes is a multistep process (Davis et al., 2007). However, a bEBP-bound RNAP-σ^54^ complex, proposed to be an intermediate state formed en route to the open complex, can be captured using the ATP hydrolysis transition-state analog ADP·AlF_x_ (Burrows et al., 2004; Chaney et al., 2001; Leach et al., 2006; Wigneshweraraj et al., 2005) or an ATP ground-state analog ADP·BeF (Chen et al., 2007).

**Figure 1:**
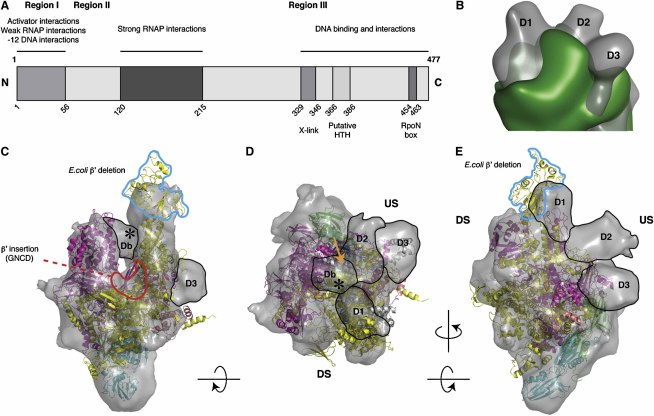
Cryo-EM Reconstructions of *E. coli* RNAP-σ^54^ Holoenzyme and RNAP with the Crystal Structure of RNAP from *Tth* Fitted (A) σ^54^ sequence and functional regions. (B) Comparison of the RNAP-σ^54^ holoenzyme reconstruction (gray) with RNAP core (green) highlights density regions corresponding to σ^54^. (C) View from downstream side into DNA binding channel with the α subunit at the bottom. Note the claws on the top and the significantly reduced density of the β′ subunit due to a sequence deletion in *E. coli* (cyan outlines). (D) View from the top into the active channel, showing the connecting density between the claws (labeled Db). The asterisk indicates where DNA loading is believed to occur, and σ^54^ densities (black outlines) D1, D2, and D3 are labeled. The orange arrow indicates the location of the σ^A^ region 3.0 helix. (E) View from β′ side shows three extra regions of σ^54^ density labeled D1, D2, and D3. α/α, blue/green; β, magenta; β′, yellow; ω, deep salmon. DS, downstream face; US, upstream face of RNAP relative to promoter DNA. *Tth* PDB ID code, 1IW7.

Although σ^70^ and σ^54^ have no obvious sequence similarity, they bind to overlapping surfaces of core RNAP (Datwyler and Meares, 2000; Wigneshweraraj et al., 2000). σ^70^ binds consensus sequences at promoter positions −35 and −10 whereas σ^54^ binds sites at positions −24 (GG) and −12 (GC), relative to the transcription start site (+1). σ^54^ is composed of three regions (Figure 1A). Region I (amino acids 1–56) has been shown to interact with bEBPs and the −12 promoter site (Bordes et al., 2003; Sasse-Dwight and Gralla, 1988b) as well as core RNAP. Region I is also required to maintain the closed complex and for the transition to an open complex (Cannon et al., 1999; Sasse-Dwight and Gralla, 1988b, 1990). Region II (amino acids 57–107) is variable and sometimes absent. Region III (amino acids 108–477) contains a number of functional modules including major core RNAP binding (residues 120–215), a DNA-interacting region (residues 329–386, previously proposed to interact with the −12 promoter region), and the C-terminal RpoN box (residues 454–463), which has been shown to bind to the −24 promoter (Burrows et al., 2003; Doucleff et al., 2007; Sasse-Dwight and Gralla, 1988a). Although σ^54^ and σ^70^ differ in their amino acid sequences and DNA recognition modes, the structural basis for their distinct properties remains unclear.

To date, structures of the bacterial core RNAP, σ^A^ holoenzyme (σ^A^ is the σ^70^ equivalent in Gram-positive bacteria), and transcription elongation complexes from either *Thermus aquaticus* (*Taq*) or *Thermus thermophilus* (*Tth*) are available at high resolution (Murakami et al., 2002a, 2002b; Tuske et al., 2005; Vassylyev et al., 2002, 2007). By contrast, structural information on the RNAP-σ^54^ holoenzyme is sparse, being limited to recent NMR structures of the σ^54^ RpoN box alone and in complex with the −24 region of DNA (Doucleff et al., 2005, 2007) and a cryo-EM reconstruction of a bEBP-bound σ^54^ (Rappas et al., 2005).

In this study, we present three-dimensional cryo-EM reconstructions of *Escherichia coli* RNAP-σ^54^ alone and in complex with the bEBP phage shock protein F (PspF). We show that the interaction of PspF with σ^54^ induces movements of distinct domains of σ^54^ within the holoenzyme. This domain reorganization appears to eliminate a steric obstruction to DNA loading and allow the repositioning of promoter DNA, thereby providing single-stranded DNA access to the active site of core RNAP.

## Results and Discussion

### Cryo-EM Reconstruction of Eσ^54^ Reveals Distinct Domains Attributable to σ^54^

The *E. coli* RNAP-σ^54^ reconstruction displays the typical claw-shaped form common to all RNAPs (Figure 1). Comparison with an independent cryo-EM reconstruction of core RNAP alone reveals three distinct regions of additional density at the top of the claw (labeled D1, D2, and D3 in Figure 1B and in Figure S1 available online). We also observe strong connecting density in the holoenzyme reconstruction between the two pincers of the claw (labeled Db in Figures 1C and 1D). These four additional regions of density are not present in the RNAP core reconstruction, and we therefore attributed them to σ^54^. Some additional differences are observed in the β′ clamp and β′ jaw domain (Figure S1), which have previously been seen to relocate upon binding of σ factors (Murakami et al., 2002b; Vassylyev et al., 2002; Wigneshweraraj et al., 2004).

The crystal structure of the RNAP core from the RNAP-σ^A^ holoenzyme structure (Protein Data Bank [PDB] ID code 1IW7) was fitted into the reconstruction using manual docking followed by automated refinement procedures (Wriggers et al., 1999) with an overall correlation coefficient of ∼0.7 (Figures 1C–1E). The two α subunits form the backbone and the β/β′ subunits form the pincers. The three distinct regions of density labeled D1, D2, and D3 are almost exclusively on the β′ subunit side and on the upstream face of RNAP. These density regions, as well as the strong bridging density labeled Db, are not accounted for by the core RNAP crystal structure, further supporting their assignment as σ^54^ (Figures 1C–1E). We note a number of differences in the core RNAP between our *E. coli* reconstruction and the crystal structures of RNAP from *Tth* (or *Taq*), reflecting the sequence variations between the *E. coli* and the *Tth*/*Taq* enzymes (Chlenov et al., 2005) (Figure 1C; Figure S2). In particular, the β′ subunit of *E. coli* RNAP has a deletion in the N-terminal region (*Tth/Taq* residues 158–451) and an insertion in the C-terminal region (residues 943 and 1130). In our reconstruction, the density corresponding to the *Tth* β′ insertion (158–451) is missing (Figure 1C, blue outline), but additional density, corresponding to the *E. coli* β′ insertion, is located between the claws (Figure 1C, red outline). Furthermore, the β subunit in *E. coli* has two insertions, one near the N terminus (residues 223–339) and the other toward the C-terminal (938–1036) end. Extra density is observed on the β side of our reconstruction (Figure S2, labeled β insert N and β insert C).

### Assignment of σ^54^ Domains within the RNAP-σ^54^ Holoenzyme

**Figure 2:**
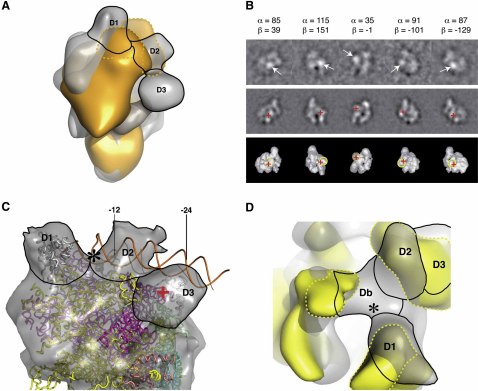
σ^54^ Domain Assignments (A) Cryo-EM reconstruction of RNAP in complex with σ^54^ lacking the C-terminal domain (RNAP-σ^54^_1–424_) (orange) displays a significantly reduced D3 density compared to the full-length RNAP-σ^54^ reconstruction (gray). D1 and D2 domains are indicated (broken lines). (B) Location of the σ^54^ C terminus by nanogold labeling. Top panel: class averages of nanogold-labeled RNAP-σ^54^ holoenzyme, with arrows pointing to the gold particles (bright circular spots); middle panel: equivalent reprojections from unlabeled RNAP-σ^54^ holoenzyme reconstruction to those of the corresponding classums (Euler angles indicated at the top) with the positions of gold particles (highest-intensity pixel in classums) marked with a red cross; bottom panel: 3D surface view along the same Euler angles as above with the D3 density regions (yellow circles) and the gold particle positions (red cross) marked. Dashed lines indicate D3 density on the opposite side of the σ^54^ holoenzyme. (C) The RNAP-σ^A^/fork junction DNA model from *Taq* (PDB ID code 1L9Z) fitted into the holoenzyme reconstruction. The asterisk indicates the location where DNA loads into the RNAP active site. The red cross indicates the center of gold particles. (D) Comparison of the RNAP-σ^54^ reconstruction (gray) with that of RNAP-σ^54^_52–477_ lacking region I (yellow). The density centered around the ^∗^ position (where DNA loading occurs) is significantly reduced, suggesting that region I in the RNAP-σ^54^ holoenzyme is located in this area. Broken lines indicate σ^54^ density regions in the σ^54^ truncation mutant reconstructions.

In order to assign the σ^54^ domains, in particular the C-terminal DNA binding domain of σ^54^ (containing the RpoN box), we obtained a cryo-EM reconstruction of RNAP in complex with σ^54^ (residues 1–424) lacking the C-terminal domain (Figure 2A). The reconstruction has a similar shape to that of RNAP-σ^54^, but differences can be seen at positions occupied by σ^54^ and the upper parts of β and β′. This is not surprising, because D3 density is an integral part of the holoenzyme complex, so the deletion of this domain is likely to affect the conformation of surrounding parts of the complex. However, density we assigned to D1 and D2 is evident in both reconstructions (Figure 2A), whereas the density corresponding to D3 is significantly reduced in the truncated σ^54^ reconstruction and cannot be accounted for by any other density, indicating that the C-terminal domain of σ^54^ is within the D3 density. To confirm this, we labeled the C-terminal 6× histidine tag of σ^54^ in the RNAP-σ^54^ holoenzyme with nanogold and imaged the particles by cryo-EM. Classification and averaging of the nanogold-labeled particles clearly reveals the site of nanogold incorporation, thereby indicating the location of the C terminus of σ^54^ (Figure 2B, top panel, arrow). The position of the highest-intensity pixel, corresponding to the nanogold particle, was plotted onto equivalent reprojections and surface views of unlabeled RNAP-σ^54^ holoenzyme (Figure 2B, middle and bottom panels, red cross). Importantly, the site of the nanogold particle colocalizes with the position of the D3 density (Figure 2B, bottom panel, yellow circle), confirming that the C terminus of σ^54^ is indeed located in the D3 density. The C-terminal domain that contains the RpoN box (residues 452–466) is responsible for binding to the −24 promoter DNA. The recent NMR structure of the *Taq* σ^54^ C-terminal domain (residues 323–389, which correspond to residues 396–466 in *E. coli*) fits well into the D3 density (Figure S3A).

In order to compare with the RNAP-σ^A^ holoenzyme, the *Tth* RNAP-σ^A^ (PDB ID code 1IW7) was fitted into the RNAP-σ^54^ holoenzyme reconstruction. When fitting the RNAP-σ^A^ as a single rigid body, we found that two of the three σ^A^ domains (region 1.1–2.4 and region 4) fit very well into two of the three σ^54^ regions of density (D1 and D3). The main core RNAP binding elements of σ^A^ (regions 1.1–2.4) fit well within the density labeled D1 (Figure 2C; Figure S3B). Residues 120–215 of σ^54^ are known to contain the major core RNAP binding determinants (Gallegos and Buck, 1999; Guo and Gralla, 1998) and were therefore assigned to this density. Region 4 of σ^A^ (responsible for −35 binding) fits well into the upstream density labeled D3 (Figure 2C; Figure S3C), proposed to be the −24 DNA binding domain of σ^54^, suggesting that the upstream promoter element binding domains of σ^54^ and σ^A^ have similar locations within their respective holoenzymes relative to the core RNAP. The remaining D2 density, along with part of the connecting density Db, could contain region I (including the −12 interacting region), region II, and part of region III of σ^54^. This is consistent with tethered iron chelate (Fe-BABE) footprinting data showing that residue 36 within region I, as well as residues 336 and 346 of region III, are in the vicinity of both the β′ rudder (310–330) and residues 525–559 of the β subunit (Wigneshweraraj et al., 2000).

To model the position of promoter DNA in the closed complex, the *Taq* RNAP-σ^A^/fork junction DNA structure (PDB ID code 1L9Z) was fitted into our holoenzyme reconstruction (Figure 2C). In this crystal structure, only minor structural changes are observed upon fork junction DNA binding (Murakami et al., 2002a, 2002b). Although we cannot discount larger structural changes in the RNAP-σ^54^ holoenzyme upon binding to promoter DNA based on our current data, we assumed that the RNAP-σ^54^ holoenzyme model presented here could represent the RNAP-σ^54^ closed complex with promoter DNA. In this model, fork junction DNA did not clash with any of the density assigned to the holoenzyme (Figure 2C), suggesting that the promoter DNA upstream of the −12 region takes a similar path in RNAP-σ^54^ to that in RNAP-σ^A^. However, σ^A^ binds to −35 and −10 promoter DNA recognition sites whereas σ^54^ binds to −24 and −12 promoter sites. In order for both σ factors to retain a similar position for binding the −12/−10 sites (where DNA strand separation nucleates), we expected the σ^54^ −24 binding domain to be located downstream of the σ^A^ −35 binding domain by a full DNA turn. However, our data show that this is not the case. In fact, the RpoN box of σ^54^ and region 4 of σ^A^ are located in similar positions in the two holoenzymes with respect to the RNAP core. This has implications for their distinct properties in initiating transcription.

Region I contains the major determinants for interacting with activator proteins (Bordes et al., 2004) and is also proposed to play an inhibitory role during transcription initiation, helping to maintain the closed complex, and is necessary for open complex formation (Chaney and Buck, 1999; Guo and Gralla, 1998). In order to locate region I of σ^54^, we obtained a cryo-EM reconstruction of the RNAP core in complex with σ^54^ lacking region I (RNAP-σ^54^[52–477]) (Figure 2D, yellow). In this reconstruction, the bridging density Db is significantly reduced and the indentation between D1, D2, and Db is absent, suggesting that region I is present at Db (Figure 2D, yellow). Interestingly, hydroxyl radical cleavage data showed that deletion of region I changes the protection pattern of σ^54^ by core RNAP. In wild-type σ^54^, the strongest protection by the core is centered on residue 397 (Casaz and Buck, 1999). When σ^54^ region I is deleted, regions 350–379 and 397–432 are strongly protected, indicating a conformational rearrangement of σ^54^ between residues 350 and 432. Comparison of the wild-type holoenzyme and RNAP-σ^54^(52–477) reconstructions reveals further changes in the σ^54^ density, notably the D2 density becomes elongated and incorporates part of D3, and there is a change in the position of the remaining D3 density (Figure 2D). These rearrangements could explain the observed changes in the footprinting data.

### The RNAP-σ^54^ Closed Complex Is Unable to Access Template DNA for Transcription

Unlike RNAP-σ^70^, the RNAP-σ^54^ holoenzyme forms a stable closed complex with promoter DNA, which is locally melted at positions −12/−11 and is unable to initiate transcription (Browning and Busby, 2004; Cannon et al., 2001; Morris et al., 1994). The strong density (Db) that connects the two pincers of RNAP (Figures 1C and 1D) is more pronounced in our reconstruction than in the RNAP-σ^A^ holoenzyme structure when filtered to the same resolution (Figure S3D). The σ^A^ region 3.0 helix sits at the upstream edge of this density (Figure 1D, arrow; Figure S3D). Transcription bubble formation and DNA loading into the RNAP active center have been proposed to occur immediately downstream of the −12 position (denoted by ^∗^ in Figures 1C and 1D), precisely where we detect strong bridging density (Db). The Db density may therefore cause a steric obstruction to the active site of RNAP and so prevent loading of DNA that has been melted proximal to the −12 position. Consequently, the presence of the Db density could explain why the RNAP-σ^54^ holoenzyme forms a conformationally stable closed complex unable to spontaneously isomerize to an open complex. In addition, based on the assignment of D3 as the RpoN box, the −12 DNA would be positioned upstream from where DNA loading into the active site is expected to occur (^∗^ in Figure 2C), based on available RNAP-σ^A^ holoenzyme structures. The position of the −12 region (Figure 2C) may help to maintain the RNAP-σ^54^ holoenzyme in a closed complex until σ^54^ is actively remodeled by a bEBP. Taken together, we argue that the inability of the RNAP active center to access the template DNA strand is a major contributing factor to the stability of the closed complex formed between RNAP-σ^54^ and promoter DNA. Activators of the σ^54^ holoenzyme are required to induce conformational changes in the holoenzyme that allow template DNA to access the active center (Burrows et al., 2008).

### The RNAP-σ^54^ Holoenzyme Undergoes Conformational Changes upon PspF Binding

In order to understand how bEBPs induce changes in the RNAP-σ^54^ holoenzyme that ultimately lead to open complex formation, we obtained a 3D reconstruction of the AAA+ domain of PspF (PspF_1–275_, which is sufficient for transcription activation; Bordes et al., 2003; Rappas et al., 2005) in complex with RNAP-σ^54^ using the ATP transition-state analog ADP·AlF_x_ to stabilize the complex.

**Figure 3:**
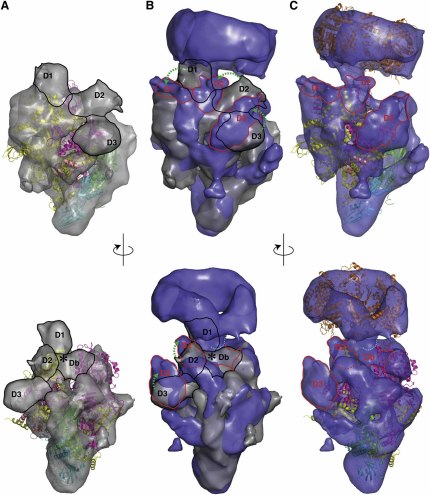
Cryo-EM Reconstruction of the Activator-Bound Complex and Comparison with the RNAP-σ^54^ Holoenzyme (A) RNAP-σ^54^ reconstruction viewed from the β′ side (top) and upstream face (bottom). (B) Overlay of the RNAP-σ^54^-PspF reconstruction and RNAP-σ^54^ reconstruction, same views as in (A). (C) RNAP-σ^54^-PspF reconstruction. Crystal structures of PspF (orange) and RNAP-σ^A^ (as in Figure 1) are fitted. Domain movements in σ^54^ between RNAP-σ^54^ (black labels) and RNAP-σ^54^-PspF (red labels) are indicated.

Importantly, the density corresponding to the core RNAP is overall very similar in this reconstruction to that of the holoenzyme reconstruction alone (Figures 3A–3C). However, some small differences are observed between the core in the two reconstructions, especially between the β and β′ pincers (Figures S4A–S4C). This is not surprising, as some flexible parts within the core are known to move during transcription initiation, such as closing of the β′ clamp domain, which could cause differences within the core density (Vassylyev et al., 2007). The clear ring-shaped density above the holoenzyme density can be assigned to the PspF_1–275_ hexamer (Figure 3C; Figure S4D). The main connecting density between PspF and the holoenzyme is located between density regions labeled D1 and D2 in the holoenzyme (Figure 3C; Figure S4E). A number of other features are worth noting. First, up to three PspF monomers could contact the holoenzyme from above the σ^54^ density. Second, PspF is positioned closer to the β′ side on the upstream face of the holoenzyme, consistent with the proposed DNA looping mechanism for contacting the closed complex (Huo et al., 2006). Finally, the site of PspF interaction lies at a considerable distance from the C-terminal domains of the RNAP α subunits (α-CTD), known interaction sites for activators that function by recruiting RNAPs (Busby and Ebright, 1999). Similar to the RNAP-σ^54^ holoenzyme reconstruction is additional density that is not accounted for by core RNAP, located adjacent to the β′ subunit toward the upstream face. We attributed this to regions of σ^54^ density D1, D2, and D3, analogous to our previous assignment (Figure 3C), although the precise locations of the σ^54^ domains cannot be determined unambiguously at this resolution. Interestingly, these density regions do not overlap with the equivalent regions in the RNAP-σ^54^ reconstruction, suggesting that σ^54^ undergoes a rearrangement with respect to core RNAP upon PspF binding (Figure 3B). When viewed from the β′ side, significant shifts are visible of all the assigned σ^54^ domains in the direction of the downstream face when compared to the RNAP-σ^54^ holoenzyme reconstruction (Figure 3B; Figures S4E and S5). This conformational reorganization is consistent with ensemble FRET measurements of RNAP-σ^54^-DNA complexes bound to the activator (Leach et al., 2006), which suggest that, upon interaction with PspF_1–275_ and ADP·AlF_x_, all σ^54^ domains move downstream toward the transcription start site at position +1.

The main connecting density between PspF and RNAP-σ^54^ in the cryo-EM reconstruction occurs at the interface of regions D1, D2, and Db (Figure 3C; Figure S4E). The “depression” between D1, D2, and Db in the RNAP-σ^54^ closed complex (Figure S4E) is now replaced by density that reaches up to the PspF hexamer (Figures 3C; Figure S4E). Our assignment of region I within this density (Figure 2D) is in agreement with the observation that PspF interacts with σ^54^ region I (Bordes et al., 2004). Therefore, σ^54^ region I is also close to where DNA is loaded into the active center of RNAP-σ^54^ (^∗^ in Figures 1D, 2C, and 2D), and could form part of the obstruction of the active site attributed to density Db. This is consistent with the observation that deletion of region I, or selected mutations within region I of σ^54^, generate “activator-bypass” forms of σ^54^ that are capable of initiating transcription from pre-melted DNA in the absence of bEBP (Chaney and Buck, 1999; Guo and Gralla, 1998). Presumably, these mutants remove the obstruction imposed by region I, and as a result allow pre-melted DNA to be loaded into the active center of RNAP-σ^54^ in the absence of activators. Hence, the comparison of the two reconstructions suggests that upon PspF binding and ATP hydrolysis, σ^54^ region I is rearranged, which leads to the loss of its inhibitory role in maintaining the closed complex. Region I also contributes to inhibitory contacts with the −12 promoter DNA (Chaney and Buck, 1999; Guo and Gralla, 1998). Changes in the interaction between region I and the −12 promoter have been observed in activator-bound promoter complexes (Burrows et al., 2004; Cannon et al., 2003; Wigneshweraraj et al., 2001), indicating that some rearrangement of region I, relative to the promoter DNA, occurs between the closed and intermediate promoter complexes. Our reconstructions support such a view, showing that region I rearranges upon interaction with PspF, therefore potentially removing both the steric block imposed by region I and modifying the inhibitory interactions with the −12 promoter.

### Promoter DNA in the Activator-Bound Complex

**Figure 4:**
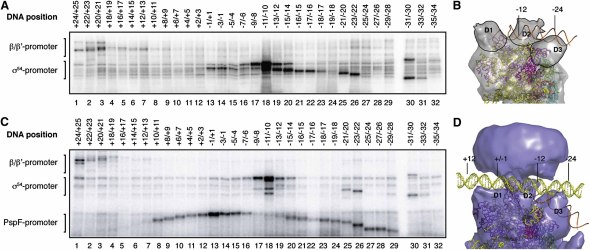
Upstream Promoter DNA Positions in the Holoenzyme and Activator-Bound Intermediate Complex The site of *p*-azidophenacyl bromide modification is labeled at the top of the gels, and migration positions of the crosslinked σ^54^ promoter, PspF promoter, and β/β′ promoter species (as identified by immunoblotting the gel with respective antibodies) are indicated. (A) Denaturing gel showing the crosslinking positions of holoenzyme promoter DNA complexes formed on the *S. meliloti nifH* promoter. (B) Side view of the holoenzyme with promoter DNA modeled in orange. (C) Denaturing gel showing the crosslinking positions of intermediate promoter DNA complexes formed on the same promoter as in (A). (D) Side view of the bEBP-bound complex with DNA modeled in (yellow). Note the relative shift in DNA (from orange in [B] to yellow in [D]) upon bEBP binding. A simple B-DNA has been used as a model and DNA positions are for indicative purposes only.

To locate promoter DNA within the activator-bound complex and to study the consequences of activator binding to the holoenzyme on promoter DNA, we analyzed the intermediate promoter complex as well as the holoenzyme promoter complex by site-directed photo-crosslinking using the well-characterized *Sinorhizobium meliloti nifH* promoter probe (Buck and Cannon, 1989, 1992). Photo-reactive (UV) promoter probes which crosslink to proteins within a 13 Å radius were constructed (Burrows et al., 2004). The denaturing gels shown in Figure 4 reveal that within the intermediate and holoenzyme complexes, interactions between the holoenzyme and upstream promoter consensus sites (−12 and −24 regions) are established mainly through σ^54^, with σ^54^ contacting DNA between positions +1 and −31 (Figure 4, lanes 13–30). Strikingly, PspF_1–275_ makes extensive crosslinks with the promoter between positions −29 and +11 (Figure 4C, lanes 8–29) relative to the transcription start site, with the greatest crosslinking efficiency observed at the −1/+1 site (Figure 4C, lane 13), possibly due to the orientation of the probe on the promoter DNA. We modeled the position of double-stranded linear DNA into the activator-bound complex on the basis that the −24 promoter region is located at density D3 (Figure 4D). This is consistent with data showing that the interaction between the RpoN box and −24 DNA remains constant between closed and open promoter complexes (Burrows et al., 2004). The location of the modeled DNA is fully consistent with our crosslinking data in that the D2 and D3 domains of σ^54^ spatially cover DNA regions from position −7 to −31 (Figures 4A and 4B), whereas the PspF_1–275_ hexamer spans DNA between positions upstream of −24 and downstream of +6 (Figures 4C and 4D). The crosslinking data show that the interactions between σ^54^ and promoter DNA are maintained around −12 and −24 upon PspF binding (as studied using the PspF/ADP·AlF complex). Our reconstructions indicate a downstream movement of σ^54^ domains is likely to occur in response to PspF binding. If σ^54^ remains bound at the −12 and −24 promoter elements, as suggested by the crosslinking data, the movement of σ^54^ would result in the DNA sliding over RNAP.

### Implications for Transcription Initiation

**Figure 5:**
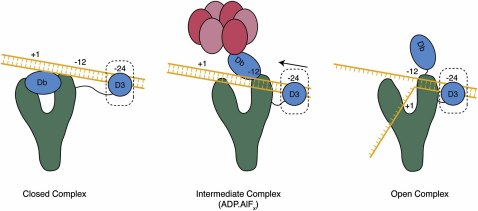
A Schematic Representation of the Proposed Relative Positions and Movements of σ^54^ Domains and Promoter DNA in the Closed, Intermediate, and Open Complexes Green, RNAP; blue, σ^54^ domains; red, PspF; orange, DNA.

Our cryo-EM reconstructions of the RNAP-σ^54^ holoenzyme and the RNAP-σ^54^-PspF_1–275_/ADP·AlF_x_ complex offer important new structural insights into the mechanism of activator-dependent DNA opening and transcription initiation. They suggest significant domain rearrangements that occur during the transcription initiation process, and provide a basis for future studies into the detailed mechanism of this process. Based on our EM reconstructions, we propose that σ^54^ region I within the RNAP-σ^54^ holoenzyme is located at, or close to, the position of DNA loading into the active site, repressing open complex formation by physically blocking entry of promoter DNA into the active site. Additionally, the −12 consensus site is held upstream of the position of DNA loading. One outcome of ATP-dependent interactions between RNAP-σ^54^ and bEBP is the conformational change of region I (and potentially parts of region III associated with −12 recognition) of σ^54^ coupled to the consequent slide of DNA toward the active site of RNAP (Figure 5). However, despite this movement, the origin of DNA melting (expected to be at position −12/−11) in the PspF-bound complex remains far from the location where DNA loading occurs, consistent with the observation that DNA is still unable to interact with the catalytic core β′/β subunits in the activator-bound complex and therefore has yet to enter the active site (Burrows et al., 2008). This configuration confirms that the activator-bound complex studied here is probably an “early” intermediate en route to the open complex (Burrows et al., 2004; Davis et al., 2007; Joly et al., 2007). Additional conformational changes must occur in both the RNAP-σ^54^ holoenzyme and the promoter DNA in order for stable open complexes to fully form (Burrows et al., 2008). This may only happen after formation of the ATP transition state, possibly involving further remodeling of σ^54^ and/or the release of PspF. We suggest that, upon completion of the ATP hydrolysis cycle, additional remodeling involves a further relocation of σ^54^ region I to allow release of PspF. This remodeling would enable the displacement of the Db density that obstructs the DNA binding channel, to allow DNA access to the active site and facilitate open complex formation (Figure 5).

## Experimental Procedures

### Protein Purification

*E. coli* PspF residues 1–275 (PspF_1–275_) and wild-type and mutant *Klebsiella pneumoniae* σ^54^ (σ^54^, σ^54^_1–424_, and σ^54^_52–477_) were cloned and purified as described (Rappas et al., 2006). Native *E. coli* RNA polymerase (RNAP, E) used for the intermediate complex formation was purified from *E. coli* MRE600 as described (Ray et al., 2003). RNAP used for the σ^54^ holoenzyme mutant and nanogold labeling analysis was prepared from B834+ cells carrying the PVS-10 plasmid (Belogurov et al., 2007) using Ni-affinity chromatography. Gel filtration was carried out using Sephacryl 300 (GE Healthcare) in buffer A (10 mM Tris-HCl [pH 8.0], 150 mM NaCl, 5% glycerol, 0.1 mM EDTA, 1 mM DTT). RNAP used for the core enzyme reconstruction was purchased from Epicenter Biotechnologies to ensure absence of any σ factors and dialyzed into buffer B (10 mM Tris-HCl [pH 8.0], 150 mM NaCl, 5% glycerol, 10 mM MgCl_2_).

### Complex Formation

RNAP-σ^54^, RNAP-σ^54^_1–424_, and RNAP-σ^54^_52–477_ were formed by incubating 25 μM RNAP core with σ^54^ in a 1:4 ratio (RNAP:σ^54^) for 5 min at 37°C. The reaction was carried out in STA buffer (25 mM Tris acetate [pH 8.0], 8 mM Mg acetate, 10 mM KCl, 1 mM DTT). Following incubation, the complex was loaded onto a Superose 6 gel-filtration column (GE Healthcare) and eluted in buffer B. The RNAP-σ^54^-PspF_1–275_ complex was formed by incubating 25 μM core RNAP with σ^54^ and PspF_1–275_ in a 1:4:12 ratio (RNAP:σ^54^:PspF_1–275_) for 5 min at 37°C. The reaction was carried out in 5× STA buffer plus 0.4 mM ADP, 5 mM NaF. After 5 min, 4 mM AlCl_3_ (final concentration) was added to the reaction mixture and incubated at 37°C for a further 10 min. The reaction mix was spun in a 100 kDa Microcon YM-100 (Millipore) centrifugal filter to remove free σ^54^ and PspF_1–275_, before loading onto a Superose 6 column. The protein was eluted in buffer B and fractions containing the full complex (purity judged by native SDS-PAGE; Figure S5) were collected and used immediately for grid preparation.

### Cryoelectron Microscopy

The homogeneity and concentration of the purified complexes was assessed using negative-stain microscopy prior to the preparation of cryo-grids. Two microliters of protein was applied to glow-discharged continuous carbon grids, blotted, and stained with 2% uranyl acetate. Samples judged to be sufficiently pure and homogeneous (Figure S5) were used to prepare cryo-grids. A 2 μl protein sample was applied to glow-discharged, holey carbon film on copper grids (400 mesh) before flash-freezing in liquid ethane using a Vitrobot (FEI). Data were collected at 50,000× magnification using a Philips CM200 FEG electron microscope operating at 200 kV under low dose conditions (10e^−^/A^2^) over a range of nominal defocus (0.5–3 μm). Images were collected on Kodak SO163 film or directly on a 4k × 4k CCD camera (F415 from Tietz Video and Imaging Processing GmbH).

### Image Processing

Images were processed with IMAGIC-5 software (van Heel et al., 2000) except where specified. Micrographs were scanned using a Nikon Coolscan 9000 ED scanner at a resolution of 4000 dpi, giving a final pixel size of 1.31 Å, or 1.76 Å if recorded directly onto the CCD. Digitized data were coarsened by a factor of 2 and particles picked interactively using the subroutine BOXER in EMAN v1.2 (Ludtke et al., 1999). Particles were extracted and CTF corrected using FindCTF2d (Grant, 2007). Initial reconstructions of the Eσ^54^ holoenzyme and the intermediate complex (Eσ^54^·PspF) were each made from ∼10,000 negatively stained particles (see Supplemental Data). The cryo-EM data sets were band-pass filtered between 170 and 7 Å. Reference-free alignment was carried out for each complex to avoid bias. Initial class averages were generated by centering the images followed by classification based on multivariate statistical analysis (MSA) and multireference alignment (MRA) (modified by Grant, 2007; see Supplemental Data) using homogeneous class averages as new references. Euler angles were assigned by angular reconstitution using the appropriate negative stain reconstruction as an anchor set. Three-dimensional reconstructions were calculated and reprojections generated from the 3D models were used as references for multiple iterations of MRA, MSA, and angular reconstitution until no further improvements in resolution were observed. Poor-quality particles were rejected throughout the refinement procedure on the basis of alignment shifts and the angular error of class averages. The final models were calculated from 10,320 particles for RNAP-σ^54^ (21 Å), 19,581 particles for RNAP-σ^54^ + PspF (21 Å), 3,008 particles for RNAP-σ^54^_1–424_ (26 Å), 4,489 particles for RNAP-σ^54^_52–477_ (24 Å), and 4,327 particles for RNAP core (23 Å). The resolution of the reconstructions was estimated by Fourier shell correlation using ½ bit resolution criteria with no masking applied (van Heel and Schatz, 2005) (Figure S6; Table S1). All 3D maps were contoured using a threshold level corresponding to the molecular mass of each complex (RNAP-σ^54^: 445 kDa; RNAP-σ^54^ + PspF: 631 kDa; RNAP-σ^54^_1–424_: 439 kDa; RNAP-σ^54^_52–477_: 438 kDa; RNAP core: 391 kDa), assuming a specific protein density of 0.83 Da/Å^3^.

### Nanogold Labeling

σ^54^ with a C-terminal 6× His tag was prepared from B834+ cells carrying the pES6 plasmid (Southern and Merrick, 2000) using Ni-affinity chromatography and eluted over a linear gradient of buffer A^Au^ (10 mM Tris HCl [pH 8.0], 300 mM NaCl, 5% glycerol) to buffer A^Au^ plus 1 M imidazole. Further purification was carried out by heparin affinity and gel filtration on a Superdex 200 column (GE healthcare) in GF buffer^Au^ (10 mM Tris HCl [pH 8.0], 150 mM NaCl, 5% glycerol) to remove traces of EDTA and DTT. σ^54^ (10 μM) (C-term-His) was mixed with 10 μM Ni-NTA nanogold (Nanoprobes) and incubated for 15 min at room temperature. Unlabeled σ^54^ (σ^54^Au) was removed by Ni-affinity chromatography (labeled protein fails to bind to the column). RNAP-σ^54^Au was formed as above and purified on a Superose 6 gel-filtration column eluted with GF buffer^Au^. Fractions corresponding to gold-labeled RNAP-σ^54^ were collected and used for electron microscopy. Grids were prepared as above under cryo conditions. Images were recorded on a Philips CM200 FEG electron microscope using a CCD camera. Particles were picked interactively using BOXER. Data were aligned against references generated from the RNAP-σ^54^ holoenzyme by projection matching, followed by MSA of each characteristic view to separate labeled from nonlabeled particles. Class averages were created containing ∼50 particles. Euler angles were assigned using an anchor set generated from the RNAP-σ^54^ holoenzyme.

### Photo-Crosslinking Assays

The *S. meliloti nifH* phosphorothiolated (QIAGEN) promoter DNA probes were derivatized with *p*-azidophenacyl bromide (APAB; Sigma) as described (Burrows et al., 2008). The modified DNA strand was then ^32^P labeled and annealed to the complementary strand as described (Wigneshweraraj et al., 2003). Photo-crosslinking reactions were conducted at 37°C in 25 mM Tris acetate (pH 8.0), 8 mM Mg acetate, 10 mM KCl, 3.5% PEG 6000 (w/v) in a total reaction volume of 10 μl as described (Burrows et al., 2004). Briefly, in a total reaction volume of 10 ml, 200 nM RNAP-σ^54^ holoenzyme assemblies were reconstituted (using 1.5 ml of 1.5 mM core RNAP and 0.5 ml of 10 mM σ^54^) at 37°C for 5 min. Modified ^32^P-labeled promoter DNA probe (20 nM) was then added to the reaction and incubated at 37°C for a further 5 min. RNAP-σ^54^ + PspF intermediate complexes were formed in situ by adding 1 μl of 10 mM ADP, 1 μl of 2 mM AlCl_3_, 1 μl of 50 mM NaF, and 1 ml of 100 μM PspF_1–275_ and incubated for 10 min at 37°C (Chaney et al., 2001). To eliminate any free core RNAP from binding to the promoter probe, RNAP-σ^54^ and RNAP-σ^54^ + PspF promoter complexes were formed in the presence of 100 ng/μl salmon sperm DNA (Oguiza et al., 1999). Reactions were then UV irradiated at 365 nm for 30 s using a UV-Stratalinker 1800 (Stratagene). The crosslinking reactions were then diluted by addition of 5 μl of 10 M urea and 5 μl of 2× SDS loading buffer (Sigma). The samples were then heated at 95°C for 3 min and 10 μl was loaded onto a 7.5% SDS-PAGE gel run at 200 V for 50 min. Gels were dried, and crosslinked protein-DNA complexes were visualized using an FLA-500 phosphorimager. Crosslinked proteins were identified using antibodies specific to *E. coli* σ^54^, β, and β′ and an anti-His tag in the case of PspF (Burrows et al., 2008).

### Fitting and Modeling

Crystal structures were fitted into the electron density manually, followed by rigid-body refinement using Situs (Wriggers et al., 1999). Transformation matrices were obtained between each of the fitted models from either the holoenzyme reconstruction or the RNAP-σ^54^ + PspF complex reconstruction. The maps were then aligned using these matrices in CCP4 (Stein et al., 1994). In the activator complex, σ^A^ region 4 bound to DNA (or the RpoN box bound to DNA) was moved manually in order to best fit into the density. The longer DNA promoter as shown in Figure 4 was obtained using the *nifH* promoter sequence (−35 to +12) via the NAB (Nucleic Acid Builder) make-na server (http://structure.usc.edu/make-na/), and positioned to best match the −24 region in the RpoN/DNA complex while satisfying the path defined by the density regions D1, D2, and D3 of σ^54^ and PspF. Difference maps were calculated using Situs (Wriggers et al., 1999) between core RNAP and the RNAP-σ^54^ holoenzyme, both filtered to 25 Å.

## Accession Numbers

Main EM maps have been deposited in the EMDB under the following accession codes: RNAP core, EMD-1568; RNAP-σ^54^, EMD-1567; and RNAP-σ^54^-PspF_1–275_, EMD-1566.

## Supplemental Data

Document S1. Nine Figures, One Table, and Supplemental Experimental Procedures
