# Supplementary material for: Organization of an Activator-Bound RNA Polymerase Holoenzyme
Source: Mol Cell. 2008 Nov 7;32(3):337–46. doi: 10.1016/j.molcel.2008.09.015 (PMC2680985; doi:10.1016/j.molcel.2008.09.015)
Supplement: Document S1. Nine Figures, One Table, and Supplemental Experimental Procedures [file mmc1.pdf]

Molecular Cell, Volume *32*

Supplemental Data

Organization of an Activator-Bound RNA Polymerase Holoenzyme

Daniel Bose, Tillmann Pape, Patricia C. Burrows, Mathieu Rappas, Siva R. Wigneshweraraj, Martin Buck, and Xiaodong Zhang

Supplemental Experimental Procedures

Brute force alignment method

The usual alignment in IMAGIC-V is carried out sequentially, i.e. first a translational alignment is done followed by a rotational alignment. Here we have used a modified brute force approach that works much better for noisy data sets, as it performs a full translational alignment at every rotation along a user specified sampling (typically ~5º). The program then searches for the highest cross correlation peak over all rotations, and finally refines both the rotational and translational parameters around the "best" rotation. This is done for each reference, and the overall best solution is taken in the end as the true alignment. Bad particles (like touching, misspicked or pieces of 'junk' on the edge of images) can readily be removed based on the maximum alignment shift and were rejected if the pixel shift was larger than half the box size at the outset of the refinement procedure. (PhD Thesis by Timothy Grant, Imperial College, 2007)

Fig S1

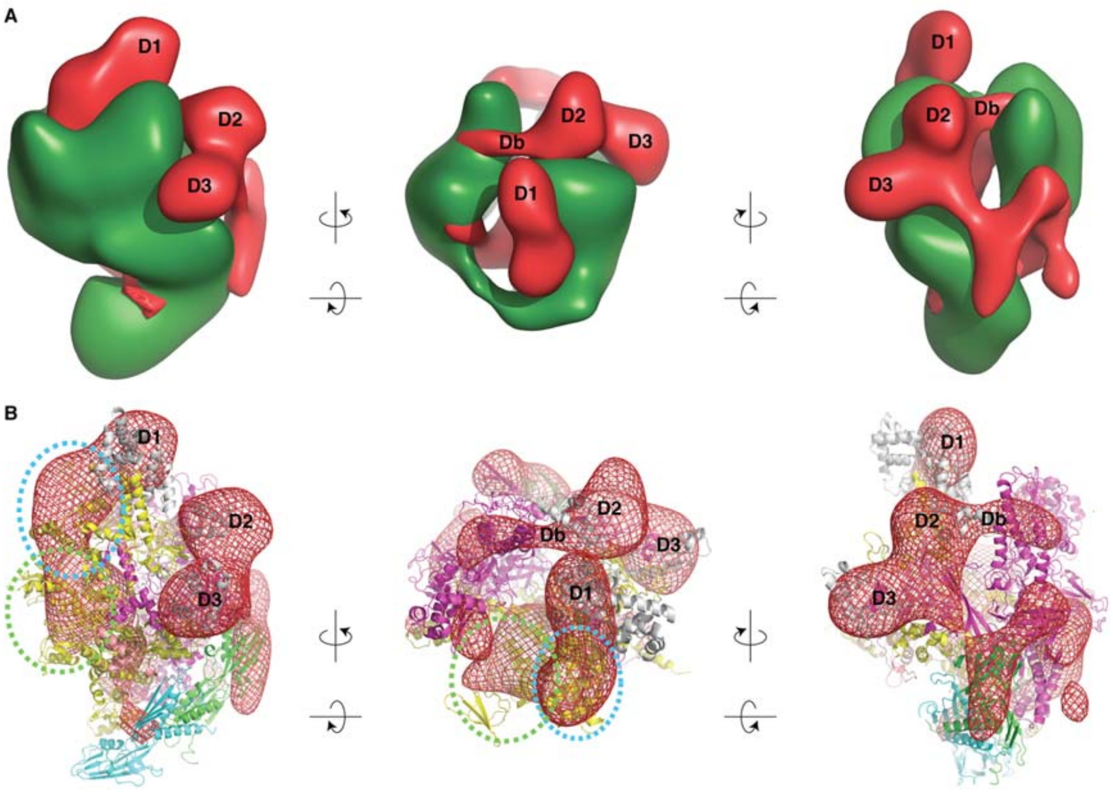

Fig S1. Difference map between core RNAP and σ^{54}-RNAP holoenzyme reconstructions. The difference map is displayed at a threshold of 4σ. A) Overlay of difference map (red) and core (green). Densities due to σ^{54} are clearly visible in the difference map. B) Overlay of difference map (red mesh) and X-ray structure of *Tth* σ^{70} RNA polymerase (1IW7). In addition to the σ^{54} densities, differences are observed in the β’clamp (blue circle) and β’-jaw (green circle). This is consistent with changes observed upon sigma binding in both crystal structures and biochemical data (Murakami et al., 2002; Vassylyev et al., 2002; Wigneshweraraj et al., 2004; Wigneshweraraj et al., 2006).

Fig S2

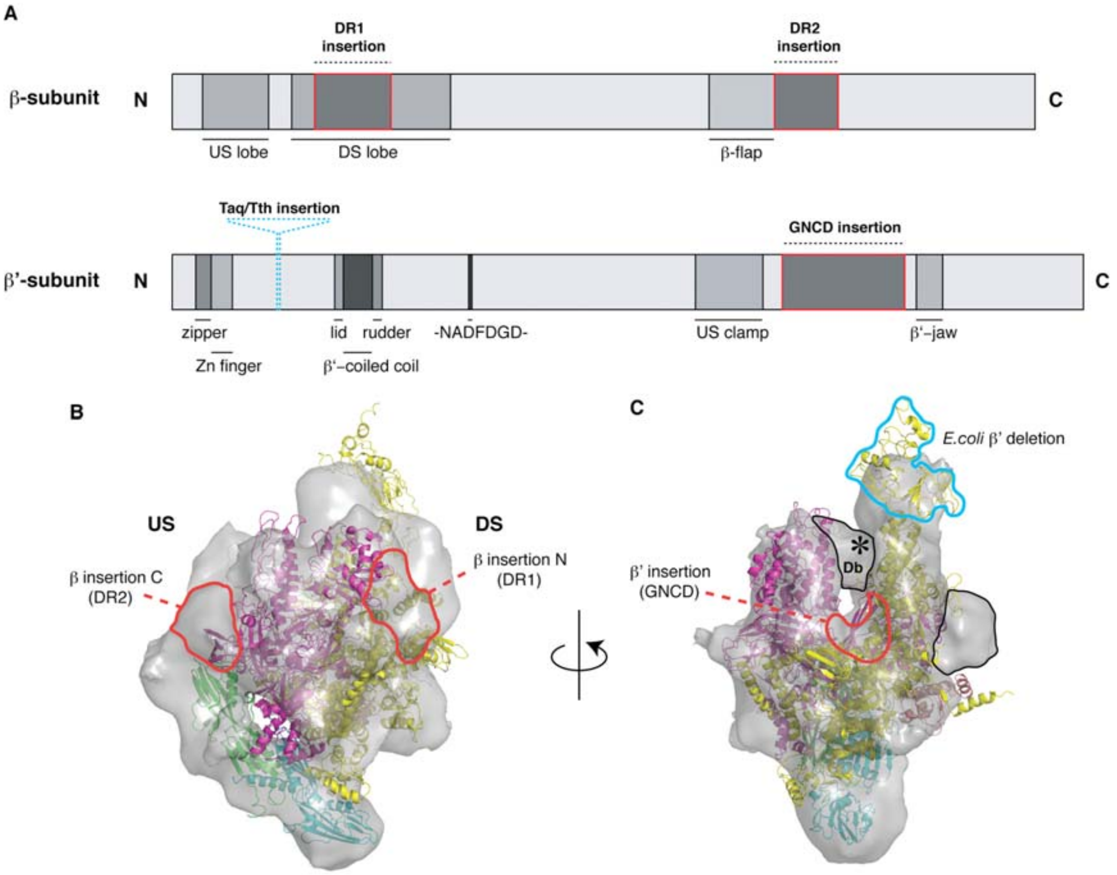

Fig S2: Insertions/deletions in *E. coli* RNAP core with respect to *Taq/Tth* RNAP core. A) β/β' subunit organisation showing insertions (red) and deletions (blue). B, C) RNAP·σ^{54} holoenzyme reconstruction with fitted *Tth* RNAP core crystal structure (1IW7) indicating the insertions (red) and deletions (blue).

Fig S3

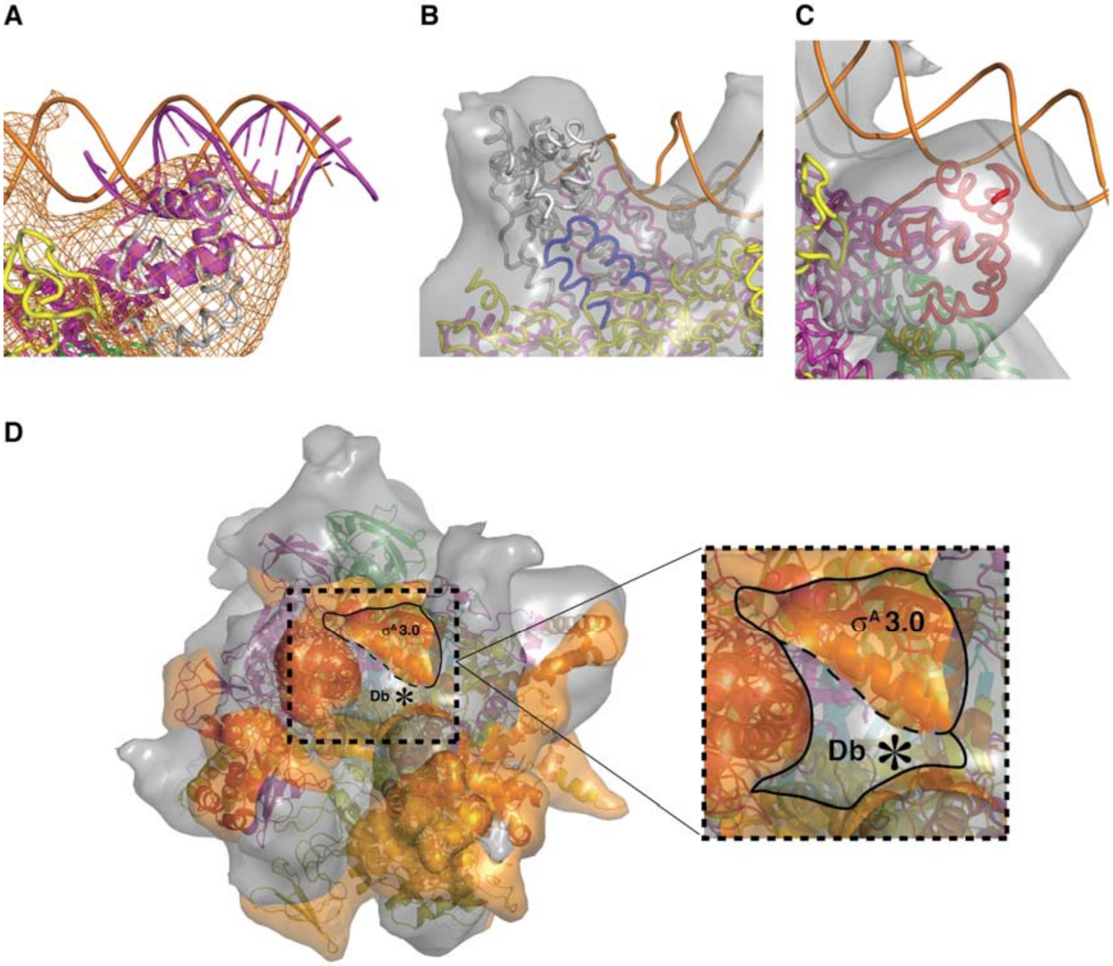

Fig S3. σ^{54} domain localisation. A) Close up of density D3 (orange mesh) with NMR structure of the σ^{54}-HTH domain including the RpoN box bound to DNA (2O91) fitted (magenta), along with σ^{A} region 4 and DNA (grey/orange). B) Close-up showing the fitting of σ^{A} regions 1.1- 2.4 into density D1. C) Close-up showing fitting of σ^{A} region 4 into density D3. D) Comparison of $E\sigma^{54}$ with $E\sigma^{70}$ holoenzyme. The $E\sigma^{70}$ holoenzyme map (orange) is calculated from the crystal structure (1IW7) in Situs (using a Gaussian filter for a target resolution of ~20 Å) and aligned with the $E\sigma^{54}$ holoenzyme reconstruction (grey). The bridging density Db and density due to σ^{A} region 3.0 are indicated, along with the position where DNA melting originates (*). Note the reduced density and upstream position of the σ^{A} region 3.0 compared to the density Db in $E\sigma^{54}$.

Fig S4

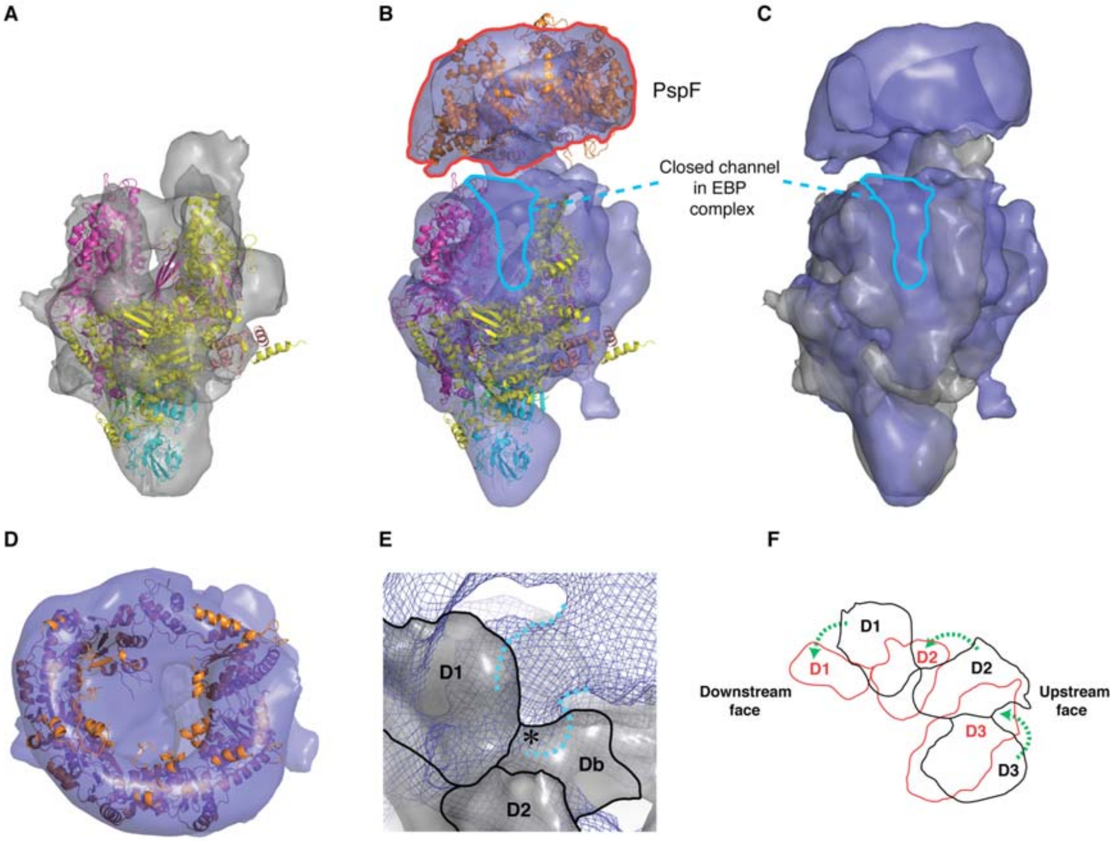

Fig S4. Domain movements of RNAP and σ^{54} in the holoenzyme upon activator binding. A) RNAP-σ^{54} reconstruction viewed from the downstream face. B) RNAP-σ^{54}-PspF$_{1\text{-}275}$ reconstruction and C) overlay of RNAP-σ^{54}-PspF reconstruction and RNAP-σ^{54} reconstruction, same views as in A). Domain movements on downstream face of RNAP are highlighted (blue outline). D) Top view of RNAP-σ^{54}-PspF$_{1\text{-}275}$ reconstruction showing the fitting of PspF$_{1\text{-}275}$ hexamer. Crystal structures of PspF$_{1\text{-}275}$ (orange) are fitted. E) Overlay of RNAP-σ^{54}-PspF reconstruction and RNAP-σ^{54} reconstruction shows connecting density between RNAP-σ^{54} and PspF (cyan outline). σ^{54} domains (black labels) and the position of DNA loading (*) are highlighted. F) Cartoon showing domain movements of σ^{54} upon PspF binding viewing from the β' side.

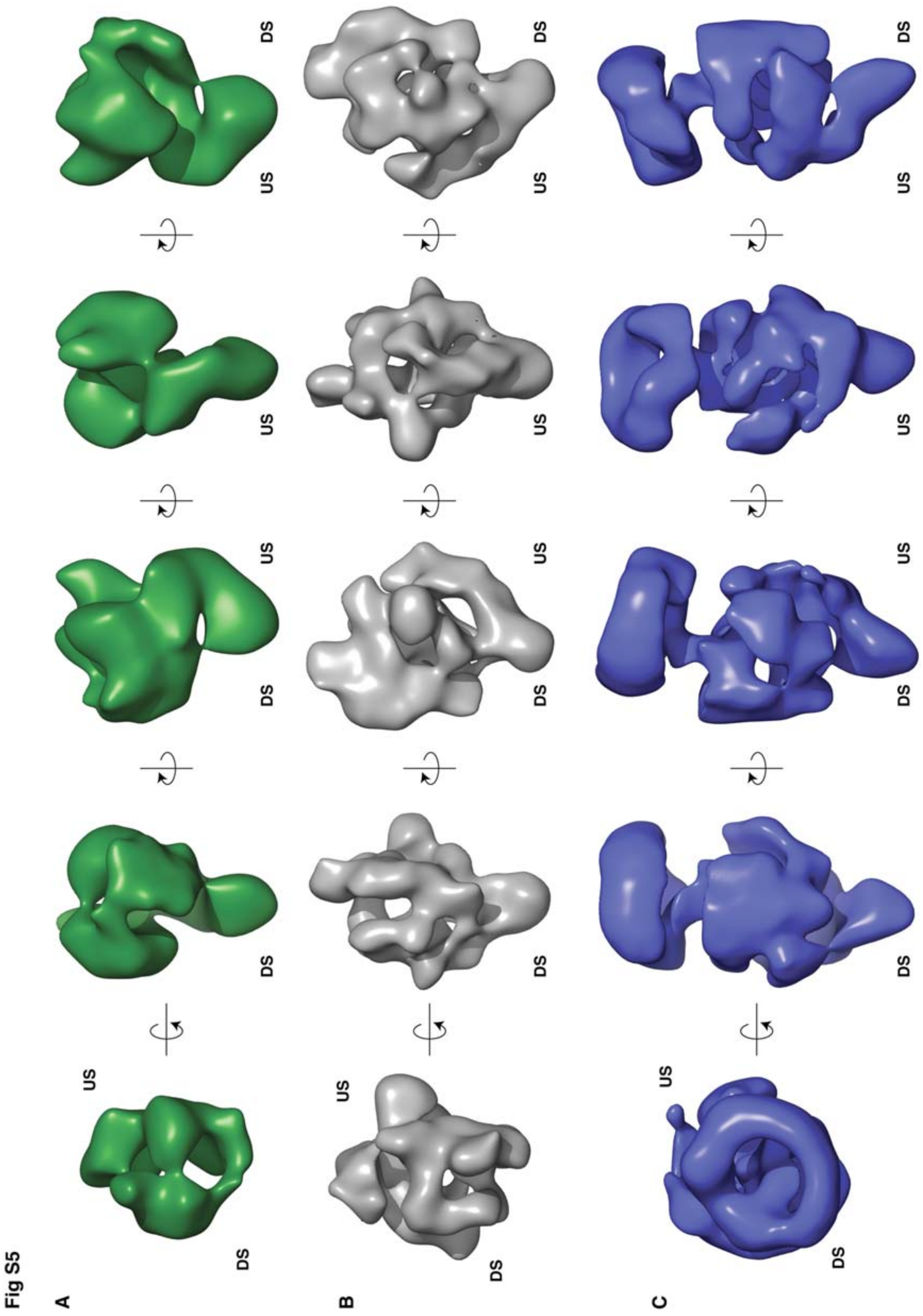

Fig S5: Comparison of representative views of: A) Core RNA polymerase (green). B) σ^{54}-holoenzyme (grey). C) Intermediate complex (RNAP-σ^{54}-PspF$^{1\text{-}275}$-ADP.AlF$_x$) (blue).

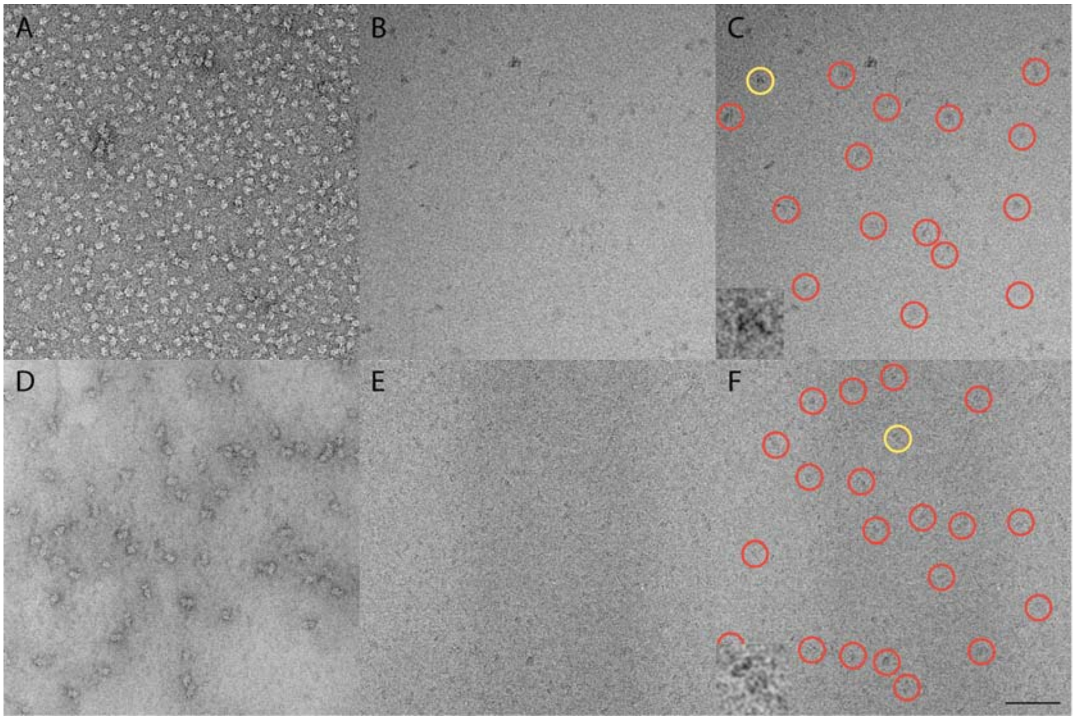

G

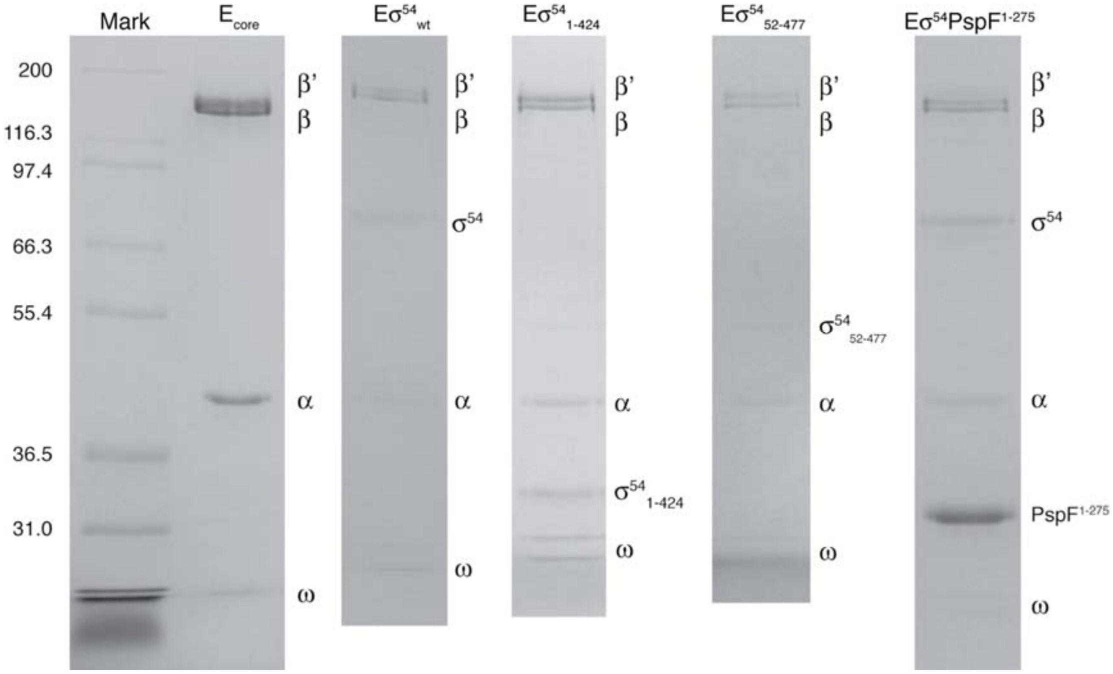

Fig S6. Homogeneity of protein complexes in this study. Representative micrographs of negatively stained (A, D) and vitrified particles (B/C, E/F) of holoenzyme (RNAP·σ^{54}) (A-C) and activator bound complex (RNAP·σ^{54}·$PspF_{1-275}$) (D-F); scale bar corresponds to 50 nm. One enlarged vitrified particle from the filtered micrograph is shown (C,F). Particles were picked from filtered micrographs and the co-ordinates used to extract from unfiltered micrographs. G) SDS PAGE gels of purified complexes (as indicated) after gel filtration showing presence of all subunits.

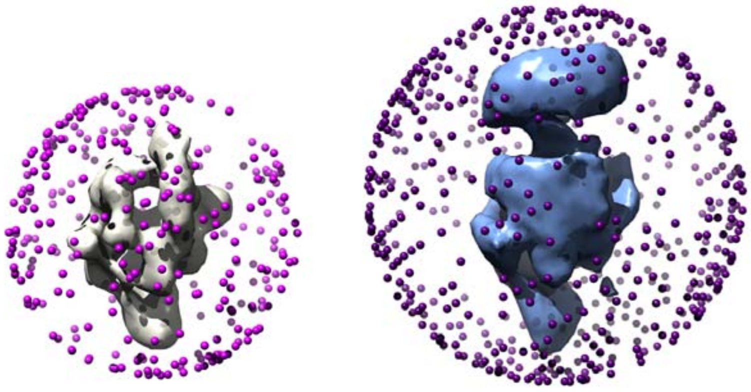

Fig S7. Euler angle distribution of the class averages for the final 3D reconstructions of the holoenzyme (RNAP-σ^{54}) and activator bound complex (RNAP-σ^{54}-$PspF_{1\text{-}275}$).

Fig. S8

A

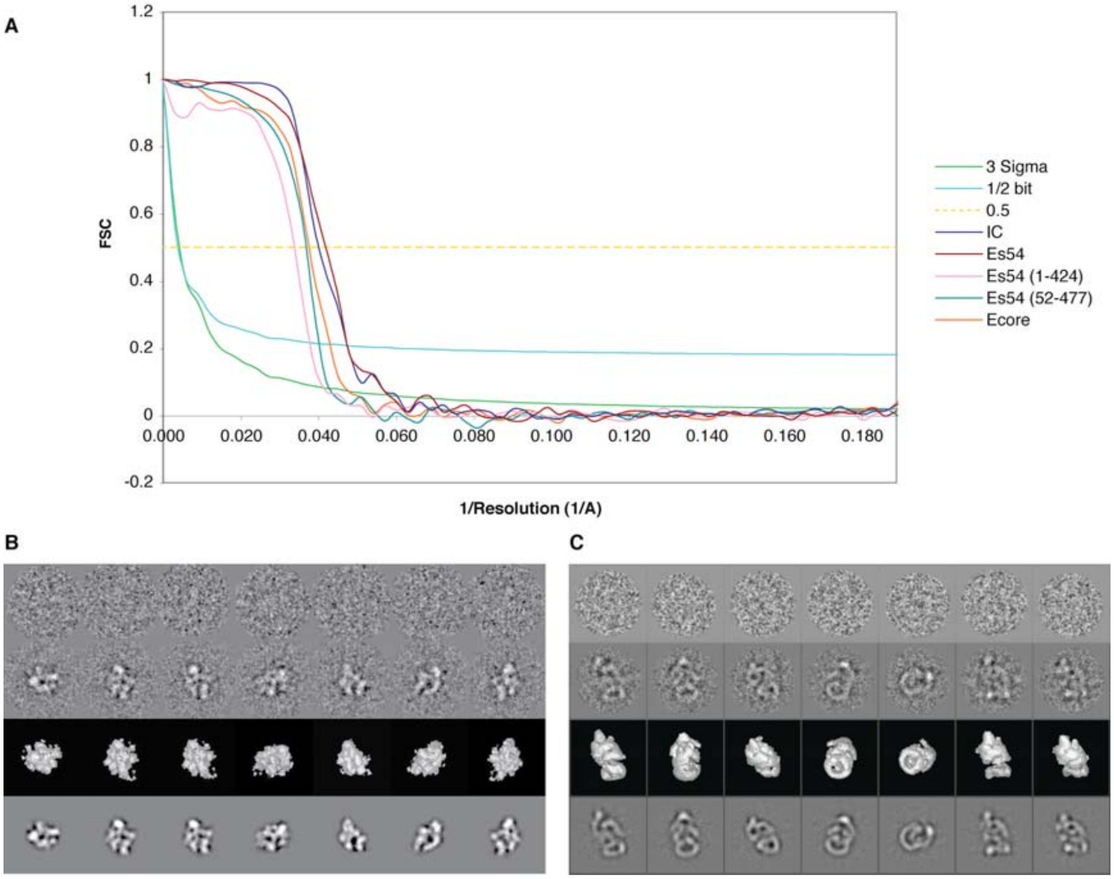

Fig S8. Overview of image processing analysis. A) Fourier Shell Correlation (FSC). FSC curves for RNAP-σ^{54}-PspF (IC, blue), Eσ^{54} (dark red), RNAP-$\sigma^{54}_{1\text{-}424}$ (pink), RNAP-$\sigma^{54}_{52\text{-}477}$ (cyan), and E core (orange) are shown, along with curves for 0.5, 1/2 bit and 3σ. B, C) Raw particles (top row), classums (2nd row), 3D model (3rd row) and reprojections from 3D model along equivalent Euler angles to classums (bottom row) for holoenzyme RNAP-σ^{54} (B) and intermediate complex RNAP-σ^{54}-PspF$_{1\text{-}275}$ (C).

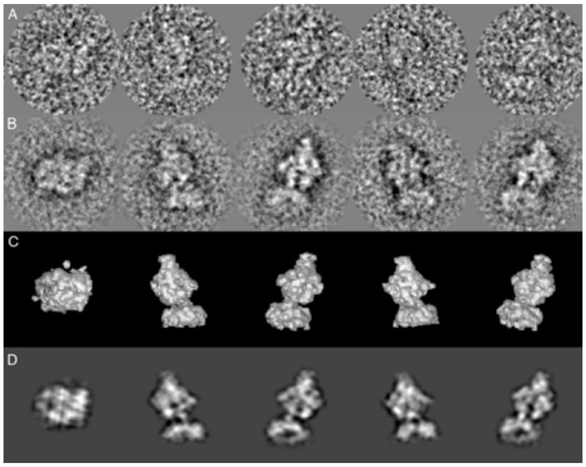

Fig S9. Initial models from negative stain data. A reference-free alignment approach was used to determine initial 3D reconstructions of holoenzyme (RNAP-σ^{54}) and activator-bound complex (RNAP-σ^{54}-PspF$_{1-275}$). Individual images (A) were centered, followed by multivariate statistical data compression and automatic classification to obtain class averages (B; 'characteristic views'). The relative orientation of class averages was determined by angular reconstitution starting with arbitrary values for the best class averages (van Heel et al., 2000). Orientations of class averages were refined iteratively against the three-dimensional model (C) obtained from the classes (B) with the lowest errors in the reconstruction compared to the reprojections from the 3D model (D). These initial reconstructions allowed us to assess and to familiarize ourselves with the samples and were further only used in this study as anchor set references for the euler angle assignment to the cryo data class averages.

Table S1. Overview of the different 3D reconstructions.

Data	# selected particles by BOXER	# particles after brute force alignment	# particles included in the final 3D reconstruction (%)	# class averages included in the final 3D reconstruction	Resolution, according to ½ bit criteria (Å)
RNAP (E)	10106	8490	4327(51%)	173	23
RNAP·σ^{54} (Eσ^{54})	24095	16234	10320 (64%)	516	21
RNAP·$\sigma^{54}_{(1-424)}$ (Eσ^{54}·ΔRpoN)	8671	5214	3008(58%)	116	26
RNAP·$\sigma^{54}_{(52-477)}$ (Eσ^{54}·ΔRegionI)	11089	7603	4489(59%)	171	24
RNAP·σ^{54}·PspF$_{1-275}$ (Eσ^{54}·PspF)	39206	23423	19581(84%)	695	21

Supplemental References

Murakami, K.S., Masuda, S., and Darst, S.A. (2002). Structural basis of transcription initiation: RNA polymerase holoenzyme at 4 A resolution. Science (New York, NY *296*, 1280-1284.

Vassylyev, D.G., Sekine, S., Laptenko, O., Lee, J., Vassylyeva, M.N., Borukhov, S., and Yokoyama, S. (2002). Crystal structure of a bacterial RNA polymerase holoenzyme at 2.6 A resolution. Nature *417*, 712-719.

Wigneshweraraj, S.R., Burrows, P.C., Nechaev, S., Zenkin, N., Severinov, K., and Buck, M. (2004). Regulated communication between the upstream face of RNA polymerase and the beta' subunit jaw domain. The EMBO journal *23*, 4264-4274.

Wigneshweraraj, S.R., Savalia, D., Severinov, K., and Buck, M. (2006). Interplay between the beta' clamp and the beta' jaw domains during DNA opening by the bacterial RNA polymerase at sigma54-dependent promoters. Journal of molecular biology *359*, 1182-1195.
